# Supplementary material for: End Sequence Analysis Toolkit (ESAT) expands the extractable information from single-cell RNA-seq data
Source: Genome Res. 2016 Oct;26(10):1397–410. doi: 10.1101/gr.207902.116 (PMC5052061; doi:10.1101/gr.207902.116)
Supplement: Supplemental Material [file supp_gr.207902.116_Supp_Materials.tar.gz › ESAT-esat_v0.1/doc/html/classumms_1_1core_1_1coordinatesystem_1_1_transcriptome_space.html]

ESAT: umms.core.coordinatesystem.TranscriptomeSpace Class Reference


ESAT


- Main Page
- Related Pages
- Packages
- Classes
- Files

- Class List
- Class Index
- Class Hierarchy
- Class Members

All Classes Namespaces Files Functions Variables Enumerator Pages

- umms
- core
- coordinatesystem
- TranscriptomeSpace

Classes |
Public Member Functions |
Static Public Member Functions |
Static Public Attributes |
List of all members

umms.core.coordinatesystem.TranscriptomeSpace Class Reference

Inheritance diagram for umms.core.coordinatesystem.TranscriptomeSpace:

[legend]

Collaboration diagram for umms.core.coordinatesystem.TranscriptomeSpace:

[legend]

|  |  |
| --- | --- |
| Classes | |
| class | GeneTree |
|  | |

|  |  |
| --- | --- |
| Public Member Functions | |
|  | TranscriptomeSpace (Map< String, Collection< Gene >> chrToGenesMap) |
|  | |
|  | TranscriptomeSpace (File bamFile, String genomeSeq) |
|  | |
|  | TranscriptomeSpace (File bamFile, Map< String, Collection< Gene >> chrToGenesMap, String genomeSeq) |
|  | |
| Collection<?extends Window > | getFragment (String chr, int start, int end) |
|  | |
| Collection<?extends Window > | getFragment (Annotation annotation) |
|  | |
| Collection<?extends Window > | getOverlappingRegion (String chr, int start, int end) |
|  | |
| Iterator< Window > | getWindowIterator (Collection< Gene > baseGenes, int windowSize, int overlap) |
|  | |
| Iterator< Window > | getWindowIterator (int windowSize, String chr, int start, int end, int overlap) |
|  | |
| Iterator<?extends Window > | getWindowIterator (Annotation window, int windowSize, int overlap) |
|  | |
| Iterator< Window > | getWindowIterator (String chr, int windowSize, int overlap) |
|  | |
| Iterator< Window > | getWindowIterator (int windowSize, int overlap) |
|  | |
| TreeMap< Integer, Double > | getPositionCountMap (Gene region, AlignmentModel data) throws IOException |
|  | |
| long | getLength () |
|  | |
| long | getLength (String chr) |
|  | |
| Annotation | permuteAnnotation (Annotation a) |
|  | |
| Annotation | permuteAnnotation (Annotation a, Annotation bounds) |
|  | |
| Collection< String > | getReferenceNames () |
|  | |
| Collection<?extends Annotation > | getReferenceAnnotations () |
|  | |
| Annotation | getReferenceAnnotation (String name) |
|  | |
| Annotation | getEntireChromosome (String chrName) |
|  | |
| int | getSize (Annotation region) |
|  | |
| boolean | hasChromosome (String chr) |
|  | |
| Annotation | moveAnnotation (Annotation read, int relativeStart, Annotation gene) |
|  | |
| Collection< String > | getChromosomeNames () |
|  | |
| boolean | isValidWindow (Annotation a) |
|  | |

|  |  |
| --- | --- |
| Static Public Member Functions | |
| static TranscriptomeSpace | getTranscriptomeSpace (String bedFile) throws IOException |
|  | |

|  |  |
| --- | --- |
| Static Public Attributes | |
| static final int | PERMUTATION\_ATTEMPTS = 10 |
|  | |

## Constructor & Destructor Documentation

|  |  |  |  |  |  |
| --- | --- | --- | --- | --- | --- |
| umms.core.coordinatesystem.TranscriptomeSpace.TranscriptomeSpace | ( | Map< String, Collection< Gene >> | *chrToGenesMap* | ) |  |

Here is the caller graph for this function:

|  |  |  |  |
| --- | --- | --- | --- |
| umms.core.coordinatesystem.TranscriptomeSpace.TranscriptomeSpace | ( | File | *bamFile*, |
|  |  | String | *genomeSeq* |
|  | ) |  |  |

Builds a transcriptome space from a bam file directly This will collapse all reads into an assembly of transcripts

Parameters
:   |  |  |
    | --- | --- |
    | bamFile | Alignment file |
    | genomeSeq | A fasta file with the reference sequence |

|  |  |  |  |
| --- | --- | --- | --- |
| umms.core.coordinatesystem.TranscriptomeSpace.TranscriptomeSpace | ( | File | *bamFile*, |
|  |  | Map< String, Collection< Gene >> | *chrToGenesMap*, |
|  |  | String | *genomeSeq* |
|  | ) |  |  |

Builds a transcriptome space from a bam file plus a known annotation file

Parameters
:   |  |  |
    | --- | --- |
    | bamFile | The reads to build the assembly from |
    | chrToGenesMap | The known transcriptome to use |

## Member Function Documentation

|  |  |  |  |  |
| --- | --- | --- | --- | --- |
| Collection<String> umms.core.coordinatesystem.TranscriptomeSpace.getChromosomeNames | ( |  | ) |  |

Returns
:   A list of chromosome names contained in the coordinate space

Implements umms.core.coordinatesystem.CoordinateSpace.

|  |  |  |  |  |  |
| --- | --- | --- | --- | --- | --- |
| Annotation umms.core.coordinatesystem.TranscriptomeSpace.getEntireChromosome | ( | String | *chrName* | ) |  |

Get an annotation spanning an entire chromosome

Parameters
:   |  |  |
    | --- | --- |
    | chrName | The chromosome name |

Returns
:   Annotation consisting of the entire chromosome

Implements umms.core.coordinatesystem.CoordinateSpace.

|  |  |  |  |
| --- | --- | --- | --- |
| Collection<? extends Window> umms.core.coordinatesystem.TranscriptomeSpace.getFragment | ( | String | *chr*, |
|  |  | int | *start*, |
|  |  | int | *end* |
|  | ) |  |  |

Return a fragment between start and end on chr

Implements umms.core.coordinatesystem.CoordinateSpace.

Here is the caller graph for this function:

|  |  |  |  |  |  |
| --- | --- | --- | --- | --- | --- |
| Collection<? extends Window> umms.core.coordinatesystem.TranscriptomeSpace.getFragment | ( | Annotation | *annotation* | ) |  |

Returns a fragment of type Window between start and end on chromosome

Parameters
:   |  |  |
    | --- | --- |
    | Annotation |  |

Returns

Implements umms.core.coordinatesystem.CoordinateSpace.

|  |  |  |  |  |
| --- | --- | --- | --- | --- |
| long umms.core.coordinatesystem.TranscriptomeSpace.getLength | ( |  | ) |  |

This will sum up the blocks ignoring the overlaps

Implements umms.core.coordinatesystem.CoordinateSpace.

|  |  |  |  |  |  |
| --- | --- | --- | --- | --- | --- |
| long umms.core.coordinatesystem.TranscriptomeSpace.getLength | ( | String | *chr* | ) |  |

Returns the total number of positions in the coordinate space For non-contiguous coordinate spaces, this will return a collapsed count such that overlapping blocks are not double counted

Parameters
:   |  |  |
    | --- | --- |
    | only | positions on a given chromosome |

Returns
:   number of positions in space

Implements umms.core.coordinatesystem.CoordinateSpace.

|  |  |  |  |
| --- | --- | --- | --- |
| Collection<? extends Window> umms.core.coordinatesystem.TranscriptomeSpace.getOverlappingRegion | ( | String | *chr*, |
|  |  | int | *start*, |
|  |  | int | *end* |
|  | ) |  |  |

Returns an interval tree over the specified transcriptome region

Implements umms.core.coordinatesystem.CoordinateSpace.

|  |  |  |  |
| --- | --- | --- | --- |
| TreeMap<Integer,Double> umms.core.coordinatesystem.TranscriptomeSpace.getPositionCountMap | ( | Gene | *region*, |
|  |  | AlignmentModel | *data* |
|  | ) |  | throws IOException |

Get map of position to of position level counts in mature transcript

Parameters
:   |  |  |
    | --- | --- |
    | region | The region |
    | data | Alignment data |

Returns
:   Map of position to count

Exceptions
:   |  |  |
    | --- | --- |
    | IOException |  |

|  |  |  |  |  |  |
| --- | --- | --- | --- | --- | --- |
| Annotation umms.core.coordinatesystem.TranscriptomeSpace.getReferenceAnnotation | ( | String | *name* | ) |  |

Parameters
:   |  |  |
    | --- | --- |
    | name |  |

Returns
:   The named reference as an annotation

Implements umms.core.coordinatesystem.CoordinateSpace.

|  |  |  |  |  |
| --- | --- | --- | --- | --- |
| Collection<? extends Annotation> umms.core.coordinatesystem.TranscriptomeSpace.getReferenceAnnotations | ( |  | ) |  |

Returns
:   The reference annotations contained in this coordinate space.

Implements umms.core.coordinatesystem.CoordinateSpace.

|  |  |  |  |  |
| --- | --- | --- | --- | --- |
| Collection<String> umms.core.coordinatesystem.TranscriptomeSpace.getReferenceNames | ( |  | ) |  |

Returns
:   A list of reference names contained in the coordinate space (e.g. chr1, chr2, etc.)

Implements umms.core.coordinatesystem.CoordinateSpace.

|  |  |  |  |  |  |
| --- | --- | --- | --- | --- | --- |
| int umms.core.coordinatesystem.TranscriptomeSpace.getSize | ( | Annotation | *region* | ) |  |

Get the size of the annotation with respect to the coordinate space

Parameters
:   |  |  |
    | --- | --- |
    | region |  |

Returns

Implements umms.core.coordinatesystem.CoordinateSpace.

Here is the call graph for this function:

Here is the caller graph for this function:

|  |  |  |  |  |  |
| --- | --- | --- | --- | --- | --- |
| static TranscriptomeSpace umms.core.coordinatesystem.TranscriptomeSpace.getTranscriptomeSpace | ( | String | *bedFile* | ) | throws IOException |

static

Here is the call graph for this function:

|  |  |  |  |
| --- | --- | --- | --- |
| Iterator<Window> umms.core.coordinatesystem.TranscriptomeSpace.getWindowIterator | ( | Collection< Gene > | *baseGenes*, |
|  |  | int | *windowSize*, |
|  |  | int | *overlap* |
|  | ) |  |  |

Get a window iterator over a collection of GeneWindows

Parameters
:   |  |  |
    | --- | --- |
    | baseGenes | The regions over which to iterate |
    | windowSize | Window size |
    | overlap | Overlap size |

Returns
:   Iterator over windows on the collection of regions

Implements umms.core.coordinatesystem.CoordinateSpace.

|  |  |  |  |
| --- | --- | --- | --- |
| Iterator<Window> umms.core.coordinatesystem.TranscriptomeSpace.getWindowIterator | ( | int | *windowSize*, |
|  |  | String | *chr*, |
|  |  | int | *start*, |
|  |  | int | *end*, |
|  |  | int | *overlap* |
|  | ) |  |  |

Iterate over windows within the defined region

Parameters
:   |  |  |
    | --- | --- |
    | windowSize |  |
    | chr |  |
    | start |  |
    | end |  |

Returns
:   returns an iterator that iterates over the region

Implements umms.core.coordinatesystem.CoordinateSpace.

|  |  |  |  |
| --- | --- | --- | --- |
| Iterator<? extends Window> umms.core.coordinatesystem.TranscriptomeSpace.getWindowIterator | ( | Annotation | *window*, |
|  |  | int | *windowSize*, |
|  |  | int | *overlap* |
|  | ) |  |  |

Returns
:   returns an iterator over an annotation sequentially

Implements umms.core.coordinatesystem.CoordinateSpace.

|  |  |  |  |
| --- | --- | --- | --- |
| Iterator<Window> umms.core.coordinatesystem.TranscriptomeSpace.getWindowIterator | ( | String | *chr*, |
|  |  | int | *windowSize*, |
|  |  | int | *overlap* |
|  | ) |  |  |

Get a window iterator from start to finish on chromosome

Implements umms.core.coordinatesystem.CoordinateSpace.

|  |  |  |  |
| --- | --- | --- | --- |
| Iterator<Window> umms.core.coordinatesystem.TranscriptomeSpace.getWindowIterator | ( | int | *windowSize*, |
|  |  | int | *overlap* |
|  | ) |  |  |

Returns
:   returns an iterator that goes from first window to last window in the coordinate space sequentially

Implements umms.core.coordinatesystem.CoordinateSpace.

|  |  |  |  |  |  |
| --- | --- | --- | --- | --- | --- |
| boolean umms.core.coordinatesystem.TranscriptomeSpace.hasChromosome | ( | String | *chr* | ) |  |

Returns true if the coordinate space contains any annotations on the queried chromosome

Parameters
:   |  |  |
    | --- | --- |
    | chr |  |

Returns

Implements umms.core.coordinatesystem.CoordinateSpace.

|  |  |  |  |  |  |
| --- | --- | --- | --- | --- | --- |
| boolean umms.core.coordinatesystem.TranscriptomeSpace.isValidWindow | ( | Annotation | *window* | ) |  |

Check if window in coordinate space and not masked

Parameters
:   |  |  |
    | --- | --- |
    | window |  |

Returns

Implements umms.core.coordinatesystem.CoordinateSpace.

Here is the call graph for this function:

|  |  |  |  |
| --- | --- | --- | --- |
| Annotation umms.core.coordinatesystem.TranscriptomeSpace.moveAnnotation | ( | Annotation | *read*, |
|  |  | int | *relativeStart*, |
|  |  | Annotation | *gene* |
|  | ) |  |  |

Moves the annotation to a new start location using the coordinate space to decide on gaps

Parameters
:   |  |  |
    | --- | --- |
    | read | the read to be moved |
    | newStartLocation | the start location of the annotation (relative to the annotation start) |
    | gene | the original transcript moving over |

Returns
:   A new annotation object in the start location reflecting the move

Here is the call graph for this function:

|  |  |  |  |  |  |
| --- | --- | --- | --- | --- | --- |
| Annotation umms.core.coordinatesystem.TranscriptomeSpace.permuteAnnotation | ( | Annotation | *a* | ) |  |

Permutes an annotation to a new location on the same reference in the given coordinate space, preserving the length of the annotation and maintaining a consistent block structure. Does not check whether the given annotation was valid in the coordinate space in the first place.

Parameters
:   |  |  |
    | --- | --- |
    | a | Annotation to permute. Will be modified. |

Returns
:   The permuted Annotation

Exceptions
:   |  |  |
    | --- | --- |
    | PermutationNotFoundException | if an annotation cannot be found |

Implements umms.core.coordinatesystem.CoordinateSpace.

Here is the call graph for this function:

|  |  |  |  |
| --- | --- | --- | --- |
| Annotation umms.core.coordinatesystem.TranscriptomeSpace.permuteAnnotation | ( | Annotation | *a*, |
|  |  | Annotation | *bounds* |
|  | ) |  |  |

Permutes an annotation to a new location within the given bounds, preserving the length of the annotation and maintaining a consistent block structure. Does not check whether the given annotation was valid in the coordinate space in the first place.

Parameters
:   |  |  |
    | --- | --- |
    | a | Annotation to permute. Will be modified. |
    | bounds | Boundaries within which the annotation will be permuted |

Returns
:   The permuted Annotation

Exceptions
:   |  |  |
    | --- | --- |
    | PermutationNotFoundException | if a permuted location cannot be found |

Implements umms.core.coordinatesystem.CoordinateSpace.

## Member Data Documentation

final int umms.core.coordinatesystem.TranscriptomeSpace.PERMUTATION\_ATTEMPTS = 10

static

---

The documentation for this class was generated from the following file:

- java/umms/core/coordinatesystem/TranscriptomeSpace.java


---

Generated on Fri Aug 15 2014 15:11:10 for ESAT by  

 1.8.7
